# Identification of Differentially Expressed Genes and Long Noncoding RNAs Associated with Parkinson's Disease

**DOI:** 10.1155/2019/6078251

**Published:** 2019-02-05

**Authors:** Lu-Mei Chi, Li-Ping Wang, Dan Jiao

**Affiliations:** ^1^Department of Neurology, China-Japan Union Hospital of Jilin University, Changchun 130033, China; ^2^Department of Ultrasound, China-Japan Union Hospital of Jilin University, Changchun 130033, China

## Abstract

**Objectives:**

This study aims to determine differentially expressed genes (DEGs) and long noncoding RNAs (lncRNAs) associated with Parkinson's disease (PD) using a microarray.

**Methods:**

We downloaded the microarray data GSE6613 from the Gene Expression Omnibus, which included 105 samples. We selected 72 samples comprising 22 healthy control blood samples and 50 PD blood samples for further analysis. Later, we used Limma to screen DEGs and differentially expressed lncRNAs (DElncRNAs) and estimated their functions by the Gene Ontology (GO). Besides, the competing endogenous RNA (ceRNA) network, including microRNAs, lncRNAs, and mRNAs, was constructed to elucidate the regulatory mechanism. Furthermore, we performed the KEGG pathway enrichment with mRNAs in the ceRNA regulatory network and constructed a final network, including pathways, mRNAs, microRNAs, and lncRNAs.

**Results:**

Overall, we obtained 394 DEGs, including 207 upregulated DEGs and 187 downregulated DEGs, and 7 DElncRNAs, including 2 upregulated DElncRNAs and 5 downregulated DElncRNAs. Insulin-like growth factor-1 receptor (IGF1R) was considerably enriched in the endocytosis pathway. In the ceRNA regulation network, IGF1R was the target of hsa-miR-133b and lncRNAs of XIST, and PART1 could also be the target of hsa-miR-133b. While the upregulated DEGs were enriched in the GO terms of the cytoskeleton, cytoskeletal part, and microtubule cytoskeleton, the downregulated DEGs were enriched in the immune response. PRKACA was markedly enriched in numerous pathways, including the MAPK and insulin signaling pathways.

**Conclusions:**

IGF1R, PRKACA, and lncRNA-XIST could be potentially involved in PD, and these diverse molecular mechanisms could support the development of the similar treatment for PD.

## 1. Introduction

Parkinson's disease (PD), a chronic and progressive neurodegenerative disease, accounts for the progressive degeneration of dopaminergic (DA) neurons in the substantia nigra [1]. Typically, PD is documented as the second leading neurodegenerative disorder, following Alzheimer's disease, and affects approximately 7 million people globally, most of which are elderly [2, 3]. As a composite disease, the occurrence of PD is attributed to multiple predisposing factors, including genetic, nongenetic, and combined effects of those factors [4, 5]. PD is almost entirely a clinical diagnosis. Lately, molecular biomarkers have been proven to be a promising clinical tool for the diagnosis of PD.

Long noncoding RNAs (lncRNAs), a major subclass of ncRNAs, are larger than 200 nucleotides in length [6] and have been reported to participate in regulating the gene expression at the transcriptional, posttranscriptional, and epigenetic levels [7, 8]. Regarding the structure, lncRNAs containing microRNAs (miRNAs) response elements could be controlled by miRNAs, thereby acting as competing endogenous RNAs (ceRNAs) to communicate with mRNAs by competing for shared miRNAs [9]. Recent experimental studies have reported lncRNAs as natural miRNA decoys in the human development and pathophysiological conditions [10]. In fact, several lncRNAs, such as ASUchl1-induced [11] and PTEN- (phosphatase and tensin homolog-) induced kinase 1 (PINK1) [12], have been reported to play an essential role in PD. Apparently, several miRNAs are related to PD. For instance, miRNA-7 inhibits neuronal apoptosis by targeting Bax and Sirt2 in PD [13] and miR-34b/c, which modulates the mitochondrial function by downregulation in PD [14].

Scherzer et al. [15] used the microarray expression profile (now available at GSE6613) to identify differentially expressed genes (DEGs) and, finally, screened ST13 as the molecular marker for PD. However, the lncRNAs profiling and the regulatory mechanism among lncRNAs, mRNAs, and miRNAs of PD remain only partially elucidated.

Using the same microarray data [15], this study aims to further screen DEGs and differentially expressed lncRNAs (DElncRNAs) between healthy control blood samples and 50 PD blood samples with linear models for the microarray data (Limma) package based on a different threshold (false discovery rate (FDR) < 0.05 and |log_2_ fold-change (FC)| > 0.5) and collect the specific genes for PD. In addition, the lncRNAs and mRNAs coexpression network, as well as the ceRNA regulatory network, was further constructed to unlock the key mechanisms underlying PD comprehensively. Reportedly, analyses based on differential statistical tests could result in different outcomes [16]; thus, we believe that some variable results might be obtained.

## 2. Materials and Methods

### 2.1. Microarray Data

We downloaded the transcription profile of GSE6613 (Affymetrix Human Genome U133 Plus 2.0 Array) [15] from the Gene Expression Omnibus (GEO; http://www.ncbi.nlm.nih.gov/geo/), which included 105 samples. We selected 72 samples comprising 22 healthy control blood samples and 50 PD blood samples for further analysis.

### 2.2. The Dataset Preprocessing and DEGs Analysis

The gene expression values were called and quantile-normalized using Oligo (version 1.41.1; http://www.bioconductor.org/packages/release/bioc/html/oligo.html) from the downloaded GEL profile. We identified mRNAs and lncRNAs in all samples and annotated using the database of the HUGO Gene Nomenclature Committee (HGNC). Then, we used the Limma package in R language to screen DEGs and DElncRNAs between PD and healthy controls [17]. In addition, Bonferroni in the multtest package was used to adjust the *P* value into the FDR [18]. We used the FDR < 0.05 and |log_2_ FC| > 0.5 as the cutoff criteria for DEGs and DElncRNAs.

### 2.3. Two-Way Clustering of DEGs and DElncRNAs

We extracted the gene expression values of considerable differentially expressed genes and lncRNAs, with which the pheatmap package in R language (version 1.0.8) was used to perform the two-way clustering based on the Euclidean distance. In addition, the online tool DAVID (the Database for Annotation, Visualization and Integrated Discovery; version 6.8; https://david.ncifcrf.gov/) was used to conduct the Gene Ontology (GO) function annotation, including Biology Process, Molecular Function, and Cellular Component. Furthermore, we screened the significantly relevant GO functions using Fisher's exact test with the criterion of *P* < 0.05.

### 2.4. lncRNAs and mRNAs Coexpression Network Construction

The coexpression analysis of lncRNAs and mRNAs was performed on the basis of the Pearson correlation coefficient using their expression levels and was visualized by Cytoscape 3.4. In addition, we analyzed the considerably relevant GO terms of the genes in the network by DAVID.

### 2.5. ceRNAs Network Analysis

#### 2.5.1. PD-Relevant miRNAs Screening

We searched the miR2Disease database (http://watson.compbio.iupui.edu:8080/miR2Disease/index.jsp) for the PD-relevant miRNAs using the keyword “Parkinson's disease.”

#### 2.5.2. lncRNA-miRNA Interaction Analysis

The interactions between lncRNAs and miRNAs were analyzed by using the starBase database (http://starbase.sysu.edu.cn/index.php ).

#### 2.5.3. mRNA-miRNA Interaction Analysis

We estimated the targets of miRNAs by using the miRanda database (http://www.microrna.org/microrna/home.do) and mapped the mRNAs that were coexpressed with lncRNAs to the targets to construct the regulatory network between miRNAs and mRNAs. Finally, we constructed the ceRNA regulatory network, which was subsequently visualized by Cytoscape 3.4.

#### 2.5.4. PD-Relevant KEGG Pathway Network Construction

We searched the Comparative Toxicogenomics Database 2017 update (http://ctd.mdibl.org/) for the PD-relevant KEGG pathways with the keyword “Parkinson's disease.” In addition, we conducted the considerably enriched KEGG pathways of the mRNAs in the ceRNA regulatory network. Finally, the overlapping pathways, mRNAs, lncRNAs, and miRNAs were used to construct the network.

## 3. Results

### 3.1. DEGs Analysis

**Figure 1:**
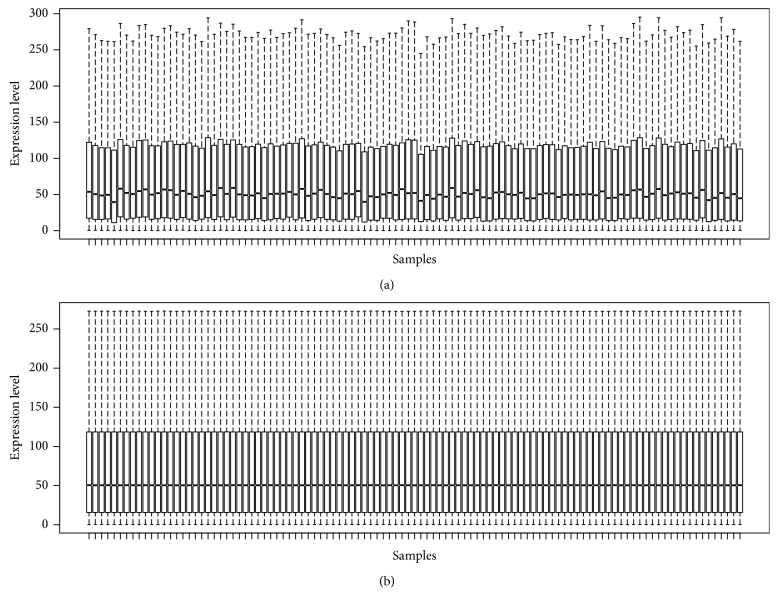
Boxplots for the data before (a) and after (b) normalization.

**Figure 2:**
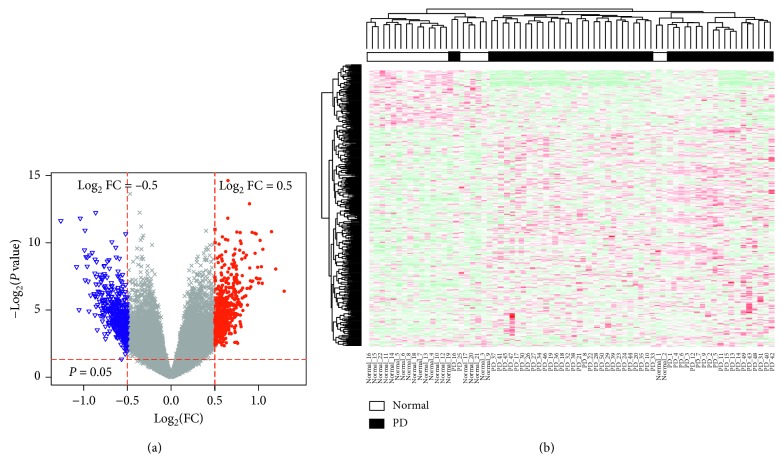
(a) The volcano plot of differentially expressed genes (DEGs); horizontal axis, log_2_(FC); vertical axis, —log_2_(*P* value). (b) The two-way clustering of DEGs between healthy control blood samples and Parkinson's disease (PD) blood samples; horizontal axis, the samples; vertical axis, DEGs between healthy control blood samples and PD blood samples.

Figure 1 shows the boxplot of the preprocessed data presenting the good normalization. We detected 19,108 mRNAs and 161 lncRNAs. Based on the microarray data analysis between PD and healthy controls by Limma, we obtained 394 DEGs, including 207 upregulated DEGs (such as insulin-like growth factor-1 receptor (IGF1R) and RPS4Y1) and 187 downregulated DEGs (such as DLG1 and PURG), and 7 DElncRNAs, including 2 upregulated DElncRNAs (LINC00302 and LINC00328) and 5 downregulated DElncRNAs (FAM215A, MCF2L-AS1, NOP14-AS1, PART1, and XIST).  Table 1 lists the top 10 markedly upregulated and downregulated DEGs, and Table 2 lists the markedly upregulated and downregulated lncRNAs. We used volcano plots for the visualization and assessment of the variation (or reproducibility) of lncRNAs and mRNAs expression between PD and healthy controls (Figure 2(a)). Furthermore, the two-way clustering revealed that lncRNA and mRNAs expression patterns between PD and healthy controls were distinguishable (Figure 2(b)).

### 3.2. GO Enrichment Analysis

**Figure 3:**
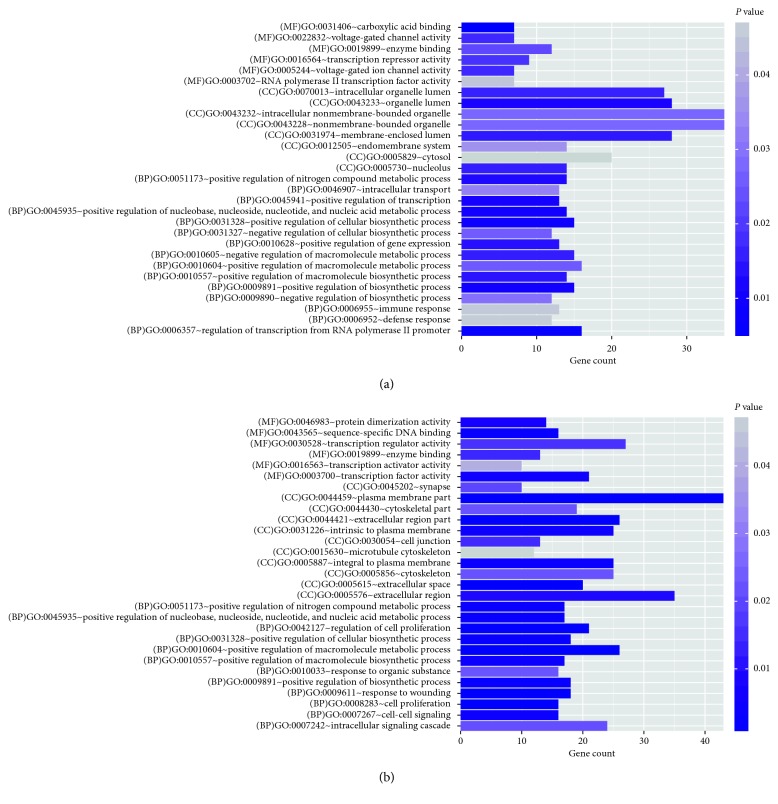
The Gene Ontology (GO) functional enrichment analyses of differentially expressed genes (DEGs) for downregulated DEGs (a) and upregulated DEGs (b).

In this study, the considerably downregulated DEGs were enriched in 29 GO terms (Table S1), such as the immune response (GO: 0006955; *P*=0.045116), positive regulation of the macromolecule metabolic process (GO: 0010604; *P*=0.025268), and positive regulation of the cellular biosynthetic process (GO: 0031328; *P*=0.008998; Figure 3(a)). However, the considerably upregulated DEGs were primarily enriched in 29 GO terms (Table S1), such as the cytoskeleton (GO: 0005856; *P*=0.022075), cytoskeletal part (GO: 0044430; *P*=0.022159), and microtubule cytoskeleton (GO: 0015630; *P*=0.047853; Figure 3(b)).

### 3.3. Coexpression Network Construction

The coexpression network comprised 296 nodes (7 lncRNAs and 289 mRNAs) and 459 coexpression pairs (440 for positive relations and 19 for negative relations; Figure S1). In addition, the mRNAs in the network were enriched in 37 GO terms, including positive regulation of the macromolecule metabolic process (GO: 0010604; *P*=1.00*E* − 04) and cell proliferation (GO: 0008283; *P*=6.38*E* − 04; Figure S2).

### 3.4. Construction and Analysis of the ceRNA Network

**Figure 4:**
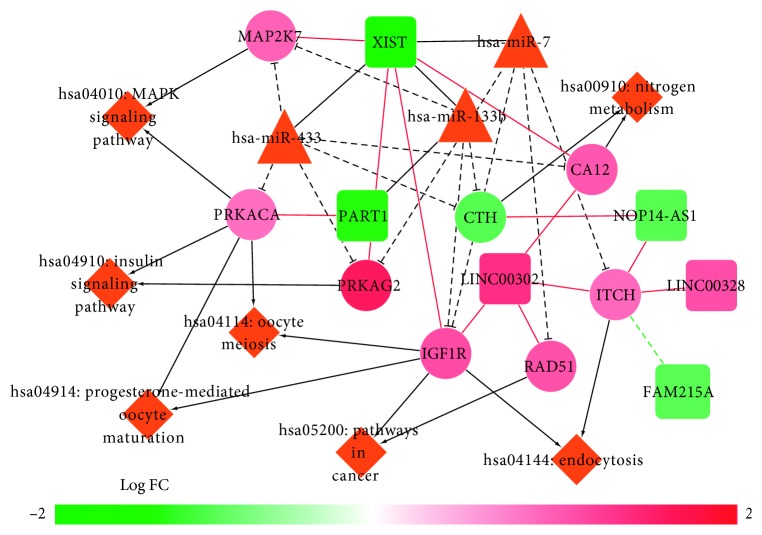
A global view of the competing endogenous RNA (ceRNA) regulatory network, including KEGG pathways in Parkinson's disease. Circles, mRNAs; triangles, microRNAs (miRNAs); squares, long noncoding RNAs (lncRNAs); black lines, the correlation between lncRNAs and miRNAs; black “T” lines, the correlation between mRNAs and miRNAs; black arrows, involvement of mRNAs in the KEGG pathways; red lines, the positive correlation between lncRNAs and mRNAs.

Based on the miR2Disease database, we screened five PD-related miRNAs, including hsa-miR-433, hsa-miR-133b, hsa-miR-7, hsa-miR-64, and hsa-miR-65. Subsequently, we identified a regulatory association among three miRNAs (hsa-miRNA-7, hsa-miRNA-133b, and hsa-miRNA-433) and two lncRNAs (XIST and PART1) according to starBase. In addition, 76 pairs of miRNAs-mRNAs were detected. Finally, the ceRNA regulatory network, including 65 nodes (3 miRNAs, 7 lncRNAs, and 55 mRNAs) and 173 regulatory pairs, was constructed (Figure S3). Furthermore, the mRNAs in the ceRNA regulatory network were considerably enriched in the GnRH signaling pathway (PRKACA and MAP2K7), insulin signaling pathway (PRKAG2 and PRKACA), endocytosis (IGF1R and ITCH), and MAPK signaling pathway (PRKACA and MAP2K7). Eventually, we constructed the final regulatory network, including lncRNAs, miRNAs, mRNAs, and pathways (Figure 4).

## 4. Discussion

PD is the second leading age-related neurodegenerative disease. However, lncRNAs and genes involved in PD await elucidation. In this study, we determined that IGF1R was considerably upregulated in PD. Reportedly, PD results from the loss of DA neurons in the substantia nigra with autophagy dysfunction [19], and IGF1R could regulate autophagy, which is closed with the PI3K-Akt-mTOR signaling pathway [20]. Moreover, the IGF1R could support the estrogen neuroprotection of the substantia nigra pars compacta DA neurons in a rat model of PD [21]. For the KEGG pathway enrichment, we established that IGF1R was markedly enriched in the pathway endocytosis. Apparently, alpha-synuclein (*α*-syn) is a presynaptic protein expressed throughout the central nervous system, and it is the primary component of Lewy bodies, one of the histopathological features of PD. Furthermore, the *α*-syn could be taken by neurons through endocytosis, the evaluation of which might account for PD [22].

For DElncRNAs between PD and controls, XIST was markedly downregulated in PD. Reportedly, lncRNA-XIST is the most vital regulator of X chromosome in mammals [23]. In non-small-cell lung cancer, XIST has been reported to regulate the cell proliferation and apoptosis by modulating the MAP3K3 pathway, and the MAPK pathway was associated with the PD pathogenesis [24]. However, further investigation is warranted to validate the function of XIST in PD. In the ceRNA regulation network, IGF1R and lncRNAs were the targets of hsa-miR-133b and XIST, respectively, and PART1 could also be the target of hsa-miR-133b. Thus, XIST and PART1 could be the sponge of hsa-miR-133b and regulate the IGF1R expression. Hence, we speculated that IGF1R could be regulated by lncRNAs of XIST, and PART1 might play an essential role in PD.

According to the GO terms enrichment with DEGs, we determined that upregulated DEGs were enriched in the GO terms of the cytoskeleton, cytoskeletal part, and microtubule cytoskeleton. A previous study [25] reported that *α*-syn could induce mitochondrial dysfunction, which was an essential factor for PD through the spectrin and actin cytoskeleton [26]. In addition, downregulated DEGs were considerably enriched in the GO terms of the immune response. Moreover, *α*-syn and its modified forms could initiate the innate and adaptive immune responses, and growing evidence has revealed an essential role of the central and peripheral immune response in driving the PD initiation and progression [27]. Consequently, the cytoskeleton-related functions, as well as the immune response, were essentially involved in the PD pathogenesis.

For the KEGG pathway enrichment with DEGs in the ceRNA regulatory network, PRKACA (markedly upregulated in PD) was considerably enriched in several pathways, such as the MAPK and insulin signaling pathways. Reportedly, the P38 MAPK signaling pathway could regulate nuclear factor-κB and inducible nitric oxide synthase expressions in the substantia nigra in a mouse model of PD [28]. PRKACA encodes a catalytic subunit of protein kinase A, and a previous study proved that the increased activation of PRKACA could result in enhanced MAPK signaling [29]. In addition, increasing evidence has suggested that insulin could cross the blood-brain barrier and affect several processes in the brain, including neuronal survival and growth, DA transmission, maintenance of synapses, and pathways involved in cognition; moreover, a process analogous to the peripheral insulin resistance occurs in the brain of patients with PD, even in those without diabetes, demonstrating that the insulin signaling pathway was a potential target for PD [30]. Consequently, PRKACA could be involved in PD by modulating the MAPK and insulin signaling pathways.

In conclusion, PRKACA, IGF1R, and lncRNA-XIST could be involved in PD. However, further research is warranted to elucidate their action mechanisms in PD.

## Figures and Tables

**Table 1:** The top ten up- and downregulated DEGs between Parkinson's disease and healthy controls.

Upregulated DEGs			Downregulated DEGs		
DEGs	Log FC	*P* value	DEGs	Log FC	*P* value
RPS4Y1	1.436363	0.015519	DLG1	−1.26172	0.000313
DDX3Y	1.29367	0.022047	PURG	−1.08174	0.003411
PRKAG2	1.148714	0.00055	HLA-DQB1	−0.99125	0.00143
GLRA3	1.049017	0.001367	PDE6D	−0.98539	0.000514
PCDH7	1.042698	0.007899	ABCD2	−0.98339	0.006745
EMP1	1.032737	0.003364	ATP8A1	−0.97496	0.002029
MAGEA2B	1.000555	0.007728	ZFAND1	−0.96026	0.003311
KRT20	0.97463	0.006048	IFI27	−0.95815	0.002065
GPR68	0.972924	0.000615	SDHAF3	−0.94893	0.007156
DCT	0.960358	0.001683	PTPN20	−0.94006	0.033347

DEGs: differentially expressed genes; log FC: log fold-change.

**Table 2:** The significantly up- and downregulated DElncRNAs between Parkinson's disease and healthy controls.

DEGs	Gene symbol	*P* value	Log FC
Upregulated	LINC00302	0.0034608	0.98936611
	LINC00328	0.0405987	0.79182406

Downregulated	XIST	0.0310748	−1.05387317
	PART1	0.0083513	−0.82664446
	MCF2L-AS1	0.0353912	−0.62511684
	NOP14-AS1	0.0642817	−0.62443023
	FAM215A	0.0537756	−0.61775554

DElncRNAs: differentially expressed long noncoding RNAs; log FC: log fold-change.

## Data Availability

The data used to support the findings of this study are available from the corresponding author upon request.

## Supplementary Materials

Supplementary Materials Supplementary Table 1: the significantly enriched GO terms by the DEGs between Parkinson's disease and healthy controls. Figure S1: the coexpression network of long noncoding RNAs (lncRNAs) and mRNAs in Parkinson's disease. Circles, mRNAs; squares, lncRNAs; red lines, the positive correlation between mRNAs and lncRNAs; green dotted lines, the negative correlation between mRNAs and lncRNAs. Figure S2: the Gene Ontology functional enrichment analyses of mRNAs in the coexpression network. Figure S3: a global view of the competing endogenous RNA (ceRNA) regulatory network in Parkinson's disease. Circles, mRNAs; triangles, microRNAs (miRNAs); squares, long noncoding RNAs (lncRNAs); black lines, the correlation between lncRNAs and miRNAs; black “T” lines, the correlation between mRNAs and miRNAs; red lines, the positive correlation between lncRNAs and mRNAs.
