# Supplementary material for: Identification of Differentially Expressed Genes and Long Noncoding RNAs Associated with Parkinson's Disease
Source: Parkinsons Dis. 2019 Feb 5;2019:6078251. doi: 10.1155/2019/6078251 (PMC6379850; doi:10.1155/2019/6078251)
Supplement: Supplementary Materials — Supplementary Table 1: the significantly enriched GO terms by the DEGs between Parkinson's disease and healthy controls. Figure S1: the coexpression network of long noncoding RNAs (lncRNAs) and mRNAs in Parkinson's disease. Circles, mRNAs; squares, lncRNAs; red lines, the positive correlation between mRNAs and lncRNAs; green dotted lines, the negative correlation between mRNAs and lncRNAs. Figure S2: the Gene Ontology functional enrichment analyses of mRNAs in the coexpression network. Figure S3: a global view of the competing endogenous RNA (ceRNA) regulatory network in Parkinson's disease. Circles, mRNAs; triangles, microRNAs (miRNAs); squares, long noncoding RNAs (lncRNAs); black lines, the correlation between lncRNAs and miRNAs; black “T” lines, the correlation between mRNAs and miRNAs; red lines, the positive correlation between lncRNAs and mRNAs. [file 6078251.f1.pdf]

Supplementary table 1 The significantly enriched GO terms by the DEGs between Parkinson's disease and healthy control

Down regulated DEGs

**Category**	**Term**	**Count**	**P-value**
Biology Process	GO:0006357~regulation of transcription from RNA polymerase II promoter	16	0.006352
	GO:0010604~positive regulation of macromolecule metabolic process	16	0.025268
	GO:0031328~positive regulation of cellular biosynthetic process	15	0.008998
	GO:0009891~positive regulation of biosynthetic process	15	0.010142
	GO:0010605~negative regulation of macromolecule metabolic process	15	0.015735
	GO:0045935~positive regulation of nucleobase, nucleoside, nucleotide and nucleic acid metabolic process	14	0.010089
	GO:0051173~positive regulation of nitrogen compound metabolic process	14	0.012878
	GO:0010557~positive regulation of macromolecule biosynthetic process	14	0.01448
	GO:0045941~positive regulation of transcription	13	0.011384
	GO:0010628~positive regulation of gene expression	13	0.014126
	GO:0046907~intracellular transport	13	0.032927
	GO:0006955~immune response	13	0.045116
	GO:0031327~negative regulation of cellular biosynthetic process	12	0.025931
	GO:0009890~negative regulation of biosynthetic process	12	0.02965
	GO:0006952~defense response	12	0.045693
Cellular Component	GO:0043232~intracellular non-membrane-bounded organelle	35	0.027486
	GO:0043228~non-membrane-bounded organelle	35	0.027486
	GO:0043233~organelle lumen	28	0.011802
	GO:0031974~membrane-enclosed lumen	28	0.015061
	GO:0070013~intracellular organelle lumen	27	0.016207
	GO:0005829~cytosol	20	0.047625

	GO:0005730~nucleolus	14	0.015726
	GO:0012505~endomembrane system	14	0.035623
Molecular Function	GO:0019899~enzyme binding	12	0.022542
	GO:0016564~transcription repressor activity	9	0.018834
	GO:0031406~carboxylic acid binding	7	0.0042
	GO:0005244~voltage-gated ion channel activity	7	0.017279
	GO:0022832~voltage-gated channel activity	7	0.017279
	GO:0003702~RNA polymerase II transcription factor activity	7	0.044643

Up regulated DEGs

**Category**	**Term**	**Count**	**P−value**
Biology Process	GO:0010604~positive regulation of macromolecule metabolic process	26	2.17E-05
	GO:0007242~intracellular signaling cascade	24	0.021454
	GO:0042127~regulation of cell proliferation	21	9.18E-04
	GO:0009611~response to wounding	18	1.66E-04
	GO:0031328~positive regulation of cellular biosynthetic process	18	0.002876
	GO:0009891~positive regulation of biosynthetic process	18	0.003334
	GO:0045935~positive regulation of nucleobase, nucleoside, nucleotide and nucleic acid metabolic process	17	0.002772
	GO:0051173~positive regulation of nitrogen compound metabolic process	17	0.003774
	GO:0010557~positive regulation of macromolecule biosynthetic process	17	0.004378
	GO:0008283~cell proliferation	16	1.93E-04

	GO:0007267~cell-cell signaling	16	0.004731
	GO:0010033~response to organic substance	16	0.022649
Cellular Component	GO:0044459~plasma membrane part	43	3.04E-04
	GO:0005576~extracellular region	35	0.009396
	GO:0044421~extracellular region part	26	7.28E-05
	GO:0005887~integral to plasma membrane	25	0.00376
	GO:0031226~intrinsic to plasma membrane	25	0.004988
	GO:0005856~cytoskeleton	25	0.022075
	GO:0005615~extracellular space	20	2.68E-04
	GO:0044430~cytoskeletal part	19	0.022159
	GO:0030054~cell junction	13	0.014823
	GO:0015630~microtubule cytoskeleton	12	0.047853
	GO:0045202~synapse	10	0.019844
Molecular Function	GO:0030528~transcription regulator activity	27	0.016292
	GO:0003700~transcription factor activity	21	0.00589
	GO:0043565~sequence-specific DNA binding	16	0.003223
	GO:0046983~protein dimerization activity	14	0.007661
	GO:0019899~enzyme binding	13	0.014207
	GO:0016563~transcription activator activity	10	0.040439

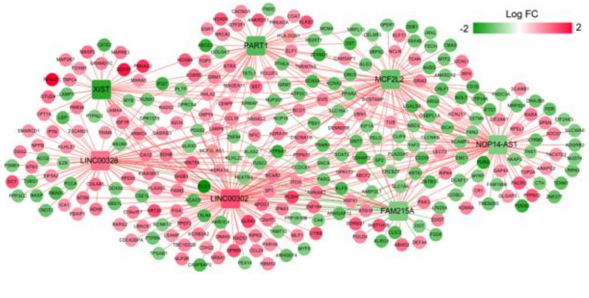
Log FC
-2
2
XIST
PART1
MCF2L2
LINC00328
LINC00302
NOP14-AS1
FAM215A

Fig S1

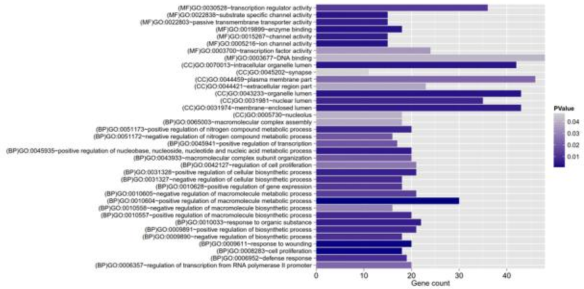
(MF)GO:0030528~transcription regulator activity
(MF)GO:0022838~substrate specific channel activity
(MF)GO:0022803~passive transmembrane transporter activity
(MF)GO:0019899~enzyme binding
(MF)GO:0015267~channel activity
(MF)GO:0005216~ion channel activity
(MF)GO:0003700~transcription factor activity
(MF)GO:0003677~DNA binding
(CC)GO:0070013~intracellular organelle lumen
(CC)GO:0045202~synapse
(CC)GO:0044459~plasma membrane part
(CC)GO:0044421~extracellular region part
(CC)GO:0043233~organelle lumen
(CC)GO:0031981~nuclear lumen
(CC)GO:0031974~membrane-enclosed lumen
(CC)GO:0005730~nucleolus
(BP)GO:0065003~macromolecular complex assembly
(BP)GO:0051173~positive regulation of nitrogen compound metabolic process
(BP)GO:0051172~negative regulation of nitrogen compound metabolic process
(BP)GO:0045941~positive regulation of transcription
(BP)GO:0045935~positive regulation of nucleobase, nucleoside, nucleotide and nucleic acid metabolic process
(BP)GO:0043933~macromolecular complex subunit organization
(BP)GO:0042127~regulation of cell proliferation
(BP)GO:0031328~positive regulation of cellular biosynthetic process
(BP)GO:0031327~negative regulation of cellular biosynthetic process
(BP)GO:0010628~positive regulation of gene expression
(BP)GO:0010605~negative regulation of macromolecule metabolic process
(BP)GO:0010604~positive regulation of macromolecule metabolic process
(BP)GO:0010558~negative regulation of macromolecule biosynthetic process
(BP)GO:0010557~positive regulation of macromolecule biosynthetic process
(BP)GO:0010033~response to organic substance
(BP)GO:0009891~positive regulation of biosynthetic process
(BP)GO:0009890~negative regulation of biosynthetic process
(BP)GO:0009611~response to wounding
(BP)GO:0008283~cell proliferation
(BP)GO:0006952~defense response
(BP)GO:0006357~regulation of transcription from RNA polymerase II promoter
0
10
20
30
40
Gene count
PValue
0.04
0.03
0.02
0.01

Fig S2

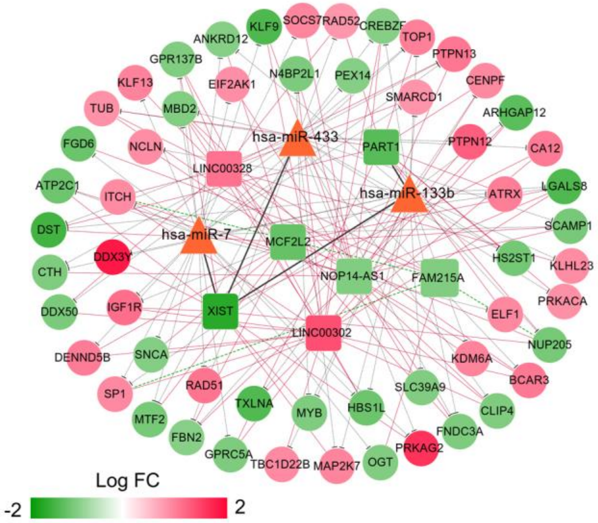
hsa-miR-433
hsa-miR-133b
hsa-miR-7
LINC00328
PART1
MCF2L2
NOP14-AS1
FAM215A
XIST
LINC00302
KLF9
SOCS7
RAD52
CREBZF
ANKRD12
TOP1
PTPN13
GPR137B
N4BP2L1
PEX14
CENPF
KLF13
EIF2AK1
SMARCD1
ARHGAP12
TUB
MBD2
FGD6
NCLN
PTPN12
CA12
ATP2C1
ITCH
ATRX
LGALS8
DST
SCAMP1
DDX3Y
HS2ST1
KLHL23
CTH
PRKACA
IGF1R
ELF1
DDX50
NUP205
DENND5B
SNCA
KDM6A
BCAR3
RAD51
SLC39A9
SP1
TXLNA
MYB
HBS1L
CLIP4
MTF2
FNDC3A
FBN2
GPRC5A
TBC1D22B
MAP2K7
OGT
PRKAG2
Log FC
-2
2

Fig S3
